# Picoeukaryotic sequences in the Sargasso Sea metagenome

**DOI:** 10.1186/gb-2008-9-1-r5

**Published:** 2008-01-07

**Authors:** Gwenael Piganeau, Yves Desdevises, Evelyne Derelle, Herve Moreau

**Affiliations:** 1 UPMC Univ Paris 06, UMR 7628, MBCE, Observatoire Océanologique, F-66651, Banyuls/mer, France; 2 CNRS, UMR 7628, MBCE, Observatoire Océanologique, F-66651, Banyuls/mer, France

## Abstract

**Background:**

With genome sequencing becoming more and more affordable, environmental shotgun sequencing of the microorganisms present in an environment generates a challenging amount of sequence data for the scientific community. These sequence data enable the diversity of the microbial world and the metabolic pathways within an environment to be investigated, a previously unthinkable achievement when using traditional approaches. DNA sequence data assembled from extracts of 0.8 μm filtered Sargasso seawater unveiled an unprecedented glimpse of marine prokaryotic diversity and gene content. Serendipitously, many sequences representing picoeukaryotes (cell size <2 μm) were also present within this dataset. We investigated the picoeukaryotic diversity of this database by searching sequences containing homologs of eight nuclear *anchor *genes that are well conserved throughout the eukaryotic lineage, as well as one chloroplastic and one mitochondrial gene.

**Results:**

We found up to 41 distinct eukaryotic scaffolds, with a broad phylogenetic spread on the eukaryotic tree of life. The average eukaryotic scaffold size is 2,909 bp, with one gap every 1,253 bp. Strikingly, the AT frequency of the eukaryotic sequences (51.4%) is significantly lower than the average AT frequency of the metagenome (61.4%). This represents 4% to 18% of the estimated prokaryotic diversity, depending on the average prokaryotic versus eukaryotic genome size ratio.

**Conclusion:**

Despite similar cell size, eukaryotic sequences of the Sargasso Sea metagenome have higher GC content, suggesting that different environmental pressures affect the evolution of their base composition.

## Abstract 2

Many sequences from picoeukaryotes were found in DNA sequence data assembled from Sargasso seawater.

## Background

Genome sequencing is becoming more and more affordable and shotgun sequencing using DNA from environmental microbial communities now provides the scientific community with a challenging amount of sequence data (see [1,2], for a review). These sequence data enable the diversity of the microbial world and the metabolic pathways within environments to be investigated [3-5], a previously unthinkable achievement when using traditional approaches, since it has been estimated that 99% of marine microorganisms can not be cultured in the laboratory [6].

Picoplankton is defined as a fraction of unicellular organisms having a cell size ranging from 0.2 to 2 or 3 μm [7] and is made up of both prokaryotic and eukaryotic cells, which can be either heterotrophic or autotrophic. The ecology of picoplankton has been intensely investigated this past decade and it now appears to play major roles in biogeochemical cycles that occur in oceans, especially in oligotrophic areas [7-9]. At present, the diversity of prokaryotes as studied mainly by PCR 16S rRNA gene based approaches [10,11], or more recently by random sequencing of filtered sea water [12], is better characterized than that of eukaryotes. For example, in samples collected from the Sargasso Sea, filtered through a pore size of 0.8 μm and randomly sequenced, Proteobacteria, Cyanobacteria and species in the CFB phylum (Cytophaga, Flavobacterium, and Bacteroides) dominated [12], while the presence of eukaryotic sequences was reported but without phylogenetic analysis. Among photosynthetic bacteria, the two genera *Prochlorococcus *and *Synechococcus *were clearly dominant, as described for many other areas [9,13].

However, although picoeukaryotes are known to be a minor component of picoplankton in terms of cell number, these organisms, at least those that are photosynthetic, are known to play a major role in primary productivity in oligotrophic areas, where they can represent up to 80% of the autotrophic biomass [7,14]. Picoeukaryotes usually have a bigger cell volume than prokaryotes, are subject to a high grazing mortality and have a higher growth rate than cyanobacteria. They can be responsible for 75% of net carbon production in some coastal areas [14]. Picoeukaryote diversity is much less well studied than its prokaryote counterpart, although some work has been done recently [15-17]. It is mainly composed of phyla such as Haptophytes, Dinoflagellates and Prasinophytes, some phylogenetic groups inside these very broad phyla still lacking cytological data [18,19]. Some quantitative studies based on *in situ *hybridization experiments showed that, among these groups, Prasinophytes apparently dominate picoeukaryotes in different oceanic areas, and, more precisely, the genus *Micromonas *[20]. However, many other species are found ubiquitously, even if they usually represent a minority of cells.

The most ambitious marine metagenomics project is the Global Ocean Survey (GOS), aiming to sequence picoplankton in many locations all over the oceans of the planet [21]. The pilot project of this study was published three years ago with samples from the Sargasso Sea [12]. The experimental design used to collect sequence data was geared largely to examining prokaryote diversity and gene content. However, some very small eukaryotes can work their way through the filtration system used (0.8 μm). This is indeed the case in the Sargasso Sea samples, where 34 18S rRNA sequences were identified but not analyzed in detail (Table S5 in [12]). Among picoeukaryote species or genera that could pass through the filtration cut off used, *Ostreococcus *is a likely candidate [12]. It is a picophytoplankton genus that belongs to Prasinophytes, a group of widespread green algae thought to have diverged very early from the ancestor of all chloroplast-containing green plants and algae. *Ostreococcus *is so far the smallest eukaryotic cell known (diameter 0.8 μm), and has the smallest currently described genome for a photosynthetic eukaryotic organism [22-24]. Here, we analyze the picoeukaryotic sequences present in the Sargasso Sea Database (SSD) to assess the sequence quality, diversity and relative abundance of these organisms and discuss the prospects of this approach for evolutionary genomics.

## Results

### Homology based approach (BLAST) versus phylogenetic tree reconstruction approach

We used sequence similarity as inferred from BLAST twice, first to retrieve eukaryotic sequences from the SSD and second to infer the taxonomic affiliation of these sequences. To retrieve the eukaryotic scaffolds from the SSD, we used a reference dataset for each gene chosen as an anchor. We used eight eukaryotic nuclear gene 'anchors', that is, well-conserved genes across the eukaryotic tree of life: 18S rRNA, 28S rRNA, and the genes encoding elongation factor 1a (EF1a), elongation factor 2 (EF2), the large subunit of RNA polymerase II (RPB1), actin, α-tubulin and β-tubulin. Since the genes we selected were well conserved among the eukaryotic lineage, we found little variation in the number of hits between the different species contained in each reference dataset. We even retrieved some prokaryotic scaffolds alongside the eukaryotic ones because of distant conservation with the protein coding genes. We are therefore confident we retrieved all eukaryotic scaffolds containing homologs to these genes using this approach. However, the taxonomic affiliation of these scaffolds as inferred from a local alignment approach has several drawbacks and has been found to be more error prone than phylogenetic based taxonomic affiliation [5]. Usually the blast best hit (BBH) against GenBank is the only way to glean information about taxonomic affiliation from most environmental sequences. The reliability of the affiliation depends on the representation of each taxonomic group in GenBank, but there is a high bias towards sequences from Metazoans in this database, with a bias towards larger organisms in general. To exemplify this, we identified no SSD scaffolds found to contain RPB1 matching with a Chlorophyta RPB1, simply because there are no Chlorophyta RPB1 genes in the GenBank protein database yet. Therefore, the taxonomic affiliation is best described for genes sequenced in a large number of species in a broad range of taxa, such as the rRNA sequences. We also checked the taxonomic affiliation by phylogenetic tree reconstruction for the rRNA sequences (see Additional data files 1 and 2 for the 28S rRNA and 18S rRNA supertrees). The taxonomic affiliation of a SSD scaffold as inferred from its BBH was found to be consistent with the tree topology for all rRNA SSD scaffolds for which phylogenetic position could be resolved, that is, for less than half of the scaffolds (Additional data files 1 and 2). However, reducing information to phylogenetic inference is too restrictive for this kind of highly fragmented sequence data. First, because most of the sequences do not contain enough sites for their phylogenetic position to be fully resolved, and second, because highly variable regions have to be discarded from the global alignment, whereas they may contain most of the information (for example, the Internal Transcribed Spacer sequences between ribosomal genes).

### Picoeukaryotic diversity of the Sargasso Sea metagenome

Depending on which gene we searched for, we retrieved 4 (EF2) to 41 (28SrRNA) distinct eukaryotic sequences from the SSD (Table 1). This is less than the 69 18S rRNA sequences reported in [12] because we analyzed the assembled sequence data deposited in GenBank, which does not contain the sequences obtained from samples 5 to 8 with larger filter sizes [25] (up to 20 μm; Table S1 in [12]). The taxonomic distribution of the sequences, as inferred from BLAST search against GenBank and phylogenetic analysis, is shown in Table 1. Despite the small number of sequences, the species diversity covered is impressive, since the five groups of the tree of eukaryotes [26] are represented for three of the eight nuclear genes (18S rRNA, RPB1, actin). The most abundant high blast score hits were found to sequences from the Dinophyceae (four out of the eight nuclear genes studied). This is consistent with previously reported marine picoeukaryotic diversity studies based on hundreds of 18S rRNA sequences from water filtered through larger pore sizes (5 and 3 μm filter pore size in [19,27], respectively). The second most abundant group belongs to the Streptophyta-Chlorophyta (green plants) group, as might be expected for samples collected from surface water.

**Table 1:** Phylogenetic distribution of the eukaryotic SSD scaffolds

		Number of SSD sequences									
		
Supergroup	Group	18S rRNA	28S rRNA	*EF1a*	*EF2*	*RPB1*	*actin*	*α-tubulin*	*β-tubulin*	*cox1*	*rbcL*
Total		38	41	11	4	30	12	13	15	11	4
Rhizaria	Cercozoa	1	-	-	-	2	1	-	-	-	-
	Polycystinea	3	1	-	-	-	-	-	-	-	-
Chromalveolates	Apicomplexa	1	5	-	1	1	-	-	-	-	-
	Ciliophora	1	-	1	-	1	-	3	3	-	-
	Dinophyceae	10* (3)	11	1	-	-	-	3	3	1	-
	Stramenopiles	1 (1)	6 (4)	2	-	-	1	1	2	2	1
Excavates	Euglenozoa	1	-	-	1	-	1	-	1	-	-
	Heterolobosea	-	-	-	-	2	-	-	-	-	-
Plantae	Chlorophyta	3 (2)	3 (3)	2	-	x	1*	1	1	4	1*
	Streptophyta	1	1	3	2*	7*	2	2*	2*	3*	-
	Rhodophyta	-	-	-	-	4	-	-	-	-	2
Unikonts	Ichthyosporea	-	1 (1)	-	-	1	-	-	-	-	x
	Arthropoda	7	7 (5)	1*	-	-	1	-	-	-	x
	Bryozoa	2	1 (1)	-	-	-	-	-	-	-	x
	Cnidaria	4	2* (1)	-	-	-	-	-	-	-	x
	Fungi	1	1	-	-	10	3	-	-	-	x
	Platyhelminthes	1	-	-	-	2	-	-	-	-	x
	Urochordata	-	1 (1)	1	-	-	1	1	1	1	x
Unknown		1 (32)	1 (25)	-	-	-	1	-	2	-	-

The number of scaffolds for which taxonomic affiliation was confirmed by phylogenetic analysis (Additional data files 1 and 2) is indicated in brackets. The taxonomic affiliation of the largest scaffold is indicated by an asterisk. The groups for which the anchor gene has no representative in the GenBank database are indicated by x.

Since the picoeukaryotic world generally comprises cells smaller than 2 to 3 μm [19,27], the available SSD enables a glimpse of the smaller part of the picoeukaryotic fraction (cell size between 0.22 and 0.8 μm). It is not surprising, therefore, that larger Prasinophytes, such as *Bathycoccus*, with a reported cell diameter around 2 μm, were not found in the data set.

We found two 18S scaffolds and one 28S scaffold matching almost perfectly with an *Ostreococcus *strain, the smallest photosynthetic picoeukaryotic known so far [23,24]. The two SSD 18S rRNA sequences do not overlap and these two sequences could thus belong to the same *Ostreococcus*, closely related to strain RCC143, consistent with previous analysis [28].

The presence of marine environmental arthropods (BBH is a marine Copepod) and Urochordate sequences (BBH is *Ciona*) was unexpected, because these organisms are usually much bigger than 0.8 μm. Marine environmental sequences from Copepods (and from Urochordate) have been previously reported in nanoplankton studies (cell size between 2 and 20 μm) but never in picoeukaryotes. Several hypotheses can be proposed to explain the presence of such sequences, one being the presence of gametes or of cell debris from larger organisms. However, even gametes are usually bigger than 0.8 μm and the DNA in cell debris is usually degraded. Another explanation could be the presence of soluble DNA fragments in the Seawater. Finally, a contamination of the filtered batch by non-filtered water cannot be totally ruled out.

Another ecologically relevant issue is the estimation of the relative abundance of phototrophic versus heterotrophic organisms among these picoeukaryotes. Assuming that all Viridiplantae and half of Dinophyceae are phototrophs [29], we nevertheless find 9.5 phototrophs out of 41, that is less than 24%. This is consistent with a higher observed diversity of heterotrophs than autotrophs in picoplankton, suggesting a complex role of heterotrophs in the microbial food web [15]. The phylogenetic analysis of the two 18S rRNA *Ostreococcus *sequences found among the SSD showed that they belong to the deep clade (cladeB in Figure 1 from [30]), even though the sea water was collected close to the surface. This observation has also been reported for *Prochlorococcus *in samples collected from a similar location [31]. Since the four Sargasso samples making up the SSD were collected during winter deep-water mixing, this may be a possible explanation for the presence of some deep water features of the SSD, as revealed by a recent study of gene content along the water column [32]. Thus, the occurrence of deep microbial strains in surface waters of the Sargasso Sea can probably be explained by frequent upwelling in this ocean area.

**Figure 1:**
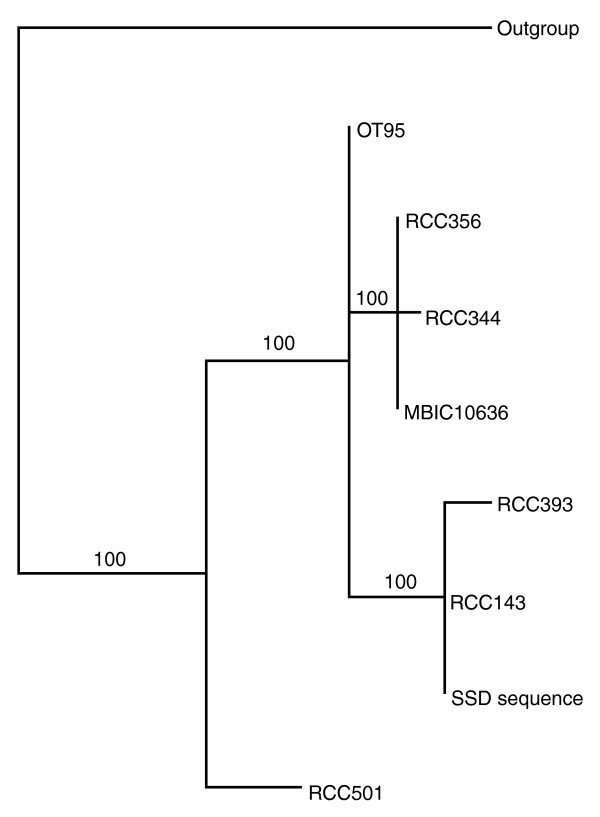
Phylogenetic position of the SSD *Ostreococcus*-like sequence as inferred from the 18S rRNA sequences in [30]. Outgroup sequence, *Bathycoccus*; OT95, *Ostreococcus tauri *(clade C); RCC356, RCC344 and MIC106, surface strains (clade A); RCC393 and RCC143, deep strains (clade B); RCC501, surface strain (clade D). Numbers on branches are support values (posterior probability).

### Picoeukaryotic diversity from other oceanic metagenomes

The SSD represents an unprecedented and yet unique sequencing effort, since it corresponds to the assembly of a total of 1.7 10^6 ^reads from four sea water samples from the Sargasso Sea [12]. In this pilot study, three other sea water samples have been sequenced in less depth and left unassembled. One of these additional samples, sample 6, used more conventional filter pore sizes to investigate the picoeukaryotic world, 0.8-3 μm, when compared to the 0.22-0.8 μm range used for three of the four SSD samples. Unfortunately, the sequencing effort of sample 6 was only 5% of the total sequencing effort realized to produce the SSD, or 29% of the smallest SSD sample. As a consequence, this sample contained far less eukaryotic material and enabled us to identify 6 (18S) to 11 (28S) additional eukaryotic paired reads, corresponding to Chromalveolates (Additional data files 1 and 2). We also screened seven additional marine metagenomes from the GOS project, corresponding to samples from seven different open ocean locations, for picoeukaryotic content. These metagenomes are part of the GOS survey [21], and sea water was filtered to collect 0.1-0.8 μm sized organisms. We found out that their picoeukaryotic content was almost negligible (from 0 to 4 reads matching a eukaryotic rRNA sequence). There are at least two reasons for this picoeukaryotic scarcity. First, the sequencing effort was much lower for these locations (4-15% of the SSD sequencing effort), thus reducing the overall diversity of the sample. Second, the collection filters used for these metagenomes were smaller (0.1 μm compared to 0.22 μm for the SSD), which also reduces the eukaryotic versus prokaryotic content. The collection filter size seems to have a major effect on picoeukaryotic sampling, since the one SSD sample collected with a 0.1 μm filter has lower picoeukaryotic content than the three other SSD samples collected with a 0.22 μm filter, despite larger sequencing depth (for example, Table S5 in [12]). Therefore, this study focuses on eukaryotic sequence diversity from the largest metagenome from the Sargasso Sea (SSD).

### Picoeukaryotic versus prokaryotic content and sequence features

We retrieved 41 distinct scaffolds containing 28S rRNA sequences and 558 distinct scaffolds containing 16S rRNA from the SSD. Assuming an equal distribution of the number of rRNA repeats in the genomes of Eukaryotes and Prokaryotes, that is, assuming that counting the number of rRNA repeats to estimate species richness is biased in the same way in both Eukaryotes and Prokaryotes, we can estimate the eukaryotic/prokaryotic species number ratio, *ρ*, equal to *ρ *= 41/558 = 7.3%. The rRNA gene copy number is known to be variable in both prokaryotes [33] and picoeukaryotes [34]. Due to the greater occurrence of duplication in eukaryotic genomes, the number of rRNA copies reached in some eukaryotic species is several orders of magnitudes higher than in prokaryotic species. Thus, the above ratio is likely to be an overestimation. The average number of different eukaryotic SSD scaffolds over the 8 nuclear genes is 20, so it seems more realistic to assume *ρ *= 20/558 = 3.7%. However, this is an underestimate because eukaryotic genomes are, on average, larger than prokaryotic ones. Assuming an equal species abundance, the probability of sequencing orthologous regions of 100 bp in two genomes of size G1 = 10 Mb, that is, of the probability of identifying two distinct species, is one order of magnitude lower than the probability of sequencing two orthologous regions of 100 bp in two genomes of size G2 = 1 Mb (equal to the ratio G1/G2). Thus, this ratio must be corrected by the difference in genome size between prokaryotic and eukaryotic organisms. However, this ratio cannot be estimated precisely, but a minimum of five seems realistic (the *Ostreococcus*/*Synechococcus *genome size ratio is 12.6/2.4 = 5.25). Thus, assuming a minimum average difference in picoeukaryotic-prokaryotic genome size of 5, *ρ *= 3.7 × 5 = 18.5%, which is consistent with recent experimental estimates of relative picoeukaryotic/prokaryotic abundance in surface coastal water [14].

Because some of the anchor genes contained the same SSD scaffolds (for 18S and 28S rRNA, α- and β-tubulin) the total number of distinct eukaryotic scaffolds for all nuclear genes is 128. The nuclear eukaryotic SSD scaffolds have two striking differences to the prokaryotic and organellar scaffolds (Table 2). The first difference is that the nuclear scaffolds are, on average, 25% shorter than the prokaryotic and organellar scaffolds (Student test between SSD scaffolds containing16S rRNA and SSD scaffolds containing 18S rRNA, *p *value < 10^-7^). The shorter length of the eukaryotic nuclear scaffolds can be explained in at least three ways. First it could solely reflect the genome size difference as explained above, since the probability of finding two overlapping sequences and, thus, larger assemblies is smaller for larger genomes. Second, it may also reflect the greater abundance of prokaryotic versus eukaryotic genomes. A greater number of prokaryotic genomes is the direct consequence of a greater number of prokaryotic cells, as estimated experimentally [14], whereas a greater number of organellar genomes could reflect a higher number of genome copies in the organelles compared to the nucleus. Our result suggests that organellar DNA may be present in more copies than nuclear DNA in picoeukaryotes, as observed in the green alga *Chlamydomonas *[35]. Third, the shorter length of eukaryotic scaffolds could also be due to different efficiencies in DNA extraction and sequencing between circular and linear DNA, or between sequences of different base composition.

**Table 2:** Comparison of the sequence features of the picoeukaryotic scaffolds retrieved from the SSD

	Number of scaffolds	Average length* (bp)	Length* of largest scaffold (Kbp)	Average distance between gap (bp)	Average AT content (%) (minimum-maximum)
All SSD	232,141	2,165	205.9	920	61.4 (16.4-99.2)
16S rRNA	558	3,942	76.1	1,103	59.9 (38.6-74.7)
18S rRNA	38	2,673	24.3	1,028	51.9 (32.5-71.1)
28S rRNA	41	1,760	4.3	880	50.5 (34.7-66.7)
*EF1a*	11	11,301	86.6	1,518	46 (33.7-67.2)
*EF2*	4	4,163	8.8	1,342	41.6 (38.5-44.6)
*RPB1*	30	1,724	2.7	862	61.9 (34.9-76.7)
*actin*	12	2,844	17.2	910	41.5 (30.1-60.3)
*α-tubulin*	13	1,986	6.7	864	48.6 (31.3-73.7)
*β-tubulin*	15	1,887	6.7	832	46.2 (30.5-73.7)
All nuclear	128	2,910	86.6	1,253	51.4 (30.1-76.7)
*cox1*	11	3,962	19.4	1,230	66.3 (58.6-70.5)
*rbcL*	4	4,173	11.3	2,033	64.7 (59.4-67.6)

*Excluding gaps.

**Figure 2:**
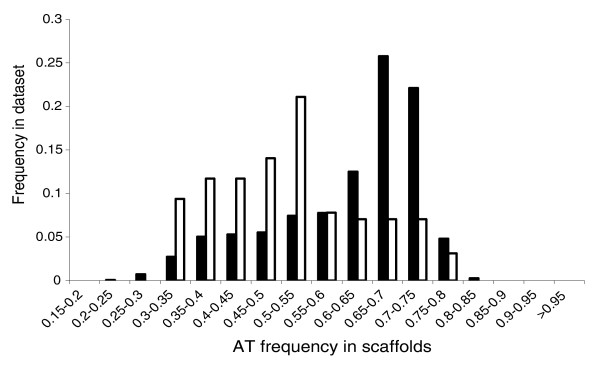
AT frequency distribution in the 128 eukaryotic SSD scaffolds retrieved (white bars) versus AT frequency distribution in the total SSD scaffolds (black bars).

The second difference is that the AT content of the SSD eukaryotic scaffolds we retrieved is much lower than the average AT content of the SSD (51.4% versus 61.4%; Student test, *p *value < 10^-15^; Figure 2). The few eukaryotic sequences we retrieved from the seven GOS open ocean locations also have a lower AT content (52.2%) than the AT content observed in these metagenomes [36]. To test whether this observation is a consequence of a GC biased anchor dataset, we compared the base composition of our anchor dataset to the average GC content in the two complete picoeukaryotic genomes of *Ostreococcus tauri *and *Cyanidioschyzon merolae*. The base composition of the eight nuclear anchor genes is actually AT biased in *O. tauri *(*n *= 8, *f*_*AT *_= 45.0% versus *n *= 7166, *f*_*AT *_= 39.6, *p *value = 0.003) and not significantly different from the average AT content of the genes in *C*.*merolae *(*n *= 8, *f*_*AT *_= 44.4, *n *= 6699, *f*_*AT *_= 44.7, *p *value = 0.79). Foerstner and colleagues [37] argued that the environment shapes the nucleotide composition of genomes because the Sargasso Sea prokaryotic sequences have a higher AT content than sequences from other environments, though the causes responsible for this compositional bias are not clear yet.

We compared the AT composition of the SSD eukaryotic sequences with the AT composition of their GenBank BBH and found no trend in average base composition differences on the alignments (exact test on the difference of AT content between each pair of sequences, *p *value = 0.93, *n *= 128); restricting the comparison to non-marine BBH was not significant either (*p *value = 0.83, *n *= 30). We also compared the AT composition of 30 of the 128 eukaryotic SSD scaffolds having a blast hit against the soil metagenome (e-value < 10^-6^) [3] and found no significant difference in base composition over the alignments (*p *value = 0.76, *n *= 30).

Shorter genome sizes and the higher cost of synthesis of G and C compared to A or T nucleotides have been invoked as possible explanations for base composition differences between genomes, because of their indirect influence on growth rate [38]. Global environmental features (nutrient availability, organism density, ecosystem complexity) may induce different pressures on growth rates and, thus, on genomic base composition [37]. This analysis suggests that base composition in picoeukaryotes is not subjected to the same selective or neutral forces as prokaryotic sequences in the Sargasso Sea.

## Discussion

We have shown that the SSD contains genomic data from at least 41 eukaryotes with cell sizes below 0.8 μm, with representatives in the five supergroups of the eukaryote tree of life. This represents 4-18% of the prokaryotic diversity of this dataset, in agreement with recent experimental estimates in surface water [14]. We cannot rule out the hypothesis that some of these sequences come from larger organisms that have contaminated some of the water samples.

Also, the assembly of environmental sequences is a great methodological challenge and erroneous assembly may lead to an over- or under-estimation of this number of distinct species. However, this is unlikely for the SSD eukaryotic data we retrieved, because the eukaryotic scaffolds are very short (of the size of the anchor genes) and most of them are 'mini-scaffolds' (consisting of a read and its mate-pair, as described in the supplementary information in [12]).

Overall, the eukaryotic scaffolds were shorter than the prokaryotic ones, which is consistent with larger genome sizes and/or lower cell numbers for picoeukaryotes, and they have a lower AT content. These sequence data contribute information for studying evolutionary genomics in marine picoeukaryotes.

Most questions in evolutionary genomics need either a complete genome or a representative subset of it. With the sequence of one organism, we can address such issues as the evolution of codon usage bias, the evolution of base composition variation, the dynamics of duplication or the dynamics of transposable elements. With several genomes sequenced from different phylogenetically related species, we can tackle similar issues but from a phylogenetic perspective (for example, which genomic process took place before or after the speciation event). We can also compare homologous sequences from two species to detect positive selection on amino-acid composition [39] or putative regulatory sequences of gene expression by phylogenetic footprinting [40,41]. However, the distinction between orthologous sequences (descending from a common ancestor by speciation) and paralogous sequences (descending from a common ancestor by duplication) is essential for evolutionary genomics [42]. This kind of information can be obtained only from a well-annotated complete genome and not from the fragmented and highly gapped environmental sequence data. However, environmental sequences such as those of the Sargasso Sea can provide precious additional data for evolutionary genomics provided that a complete genome is already available. This will soon be the case within the class of Prasinophyceae (Chlorophyta) since seven genome projects are underway: three *Ostreococcus*, three *Micromonas *and one *Bathycoccus*. For example, 13% of the 8,166 annotated coding sequences of *O. tauri*'s genome [43] match with high blast scores against the SSD (score > 105 and E-value < 10^-26^), and 41% of these scaffolds contain synteny groups with up to seven genes in the same order and orientation in both the SSD scaffold and *O*.*tauri*'s genome [41]. This metagenomic data could also be used to improve a genome assembly by bridging a gap between two genes, provided that the genomic coverage of the species is high enough in the SSD.

Another potential crucial output of metagenomes is the retrieval of new, mainly free-living, eukaryotic sequences. This could have outstanding significance for phylogenetic studies, and help to resolve the deep branches of the eukaryotic tree of life by providing sequences from missing links [16]. It is striking that the Sargasso Sea data, despite a relatively small number of different species for the same gene, contains such amazing phylogenetic spread, with representatives from the five branches of the eukaryotic tree of life [26]. Since the analysis unit of a metagenome is an assembled sequence with no more information on the organism, we need assemblies to be as long and reliable as possible to provide maximum phylogenetic information (maximum number of genes) for each organism sequenced. Unfortunately, the assembly of sequences from metagenomes is a great methodological challenge [44] and the average length of a SSD picoeukaryotic sequence is the average size of a gene, that is, around 2,000 bp for rRNA. The development of phylogenetic methods to deal with partial alignments (supertrees) enables phylogenetic inference from gapped data (for example, see references in [5,44]), thus partly overcoming this problem.

## Conclusion

Specific environmental sequencing efforts addressing more specifically picoeukaryotes are needed, with less emphasis on prokaryotes. This would enable better coverage and, thus, larger assemblies of eukaryotic genomes. The objective of the Sargasso Sea environmental sequencing was clearly to obtain prokaryotic sequences and this was done by using a very small filter porosity, sieving organisms of between 0.22 and 0.8 μm. The simplest way to improve the representation of picoeukaryotes in a metagenome would be to shift the filtration range to between 0.5 and 2 μm and increase the sequencing effort to a minimum of one million reads. This would eliminate a large fraction of the prokaryotes and would increase the proportion of picoeukaryotes present in the water sample.

## Material and methods

### Data

The SSD sequence data was retrieved from GenBank (accession number AACY01000000, Locus CH004737 to CH236877). These sequence data are the database of scaffolds not associated with any particular organism. It was obtained from samples 1-4, prefiltered through 0.8 μm and collected on one 0.1 and three 0.22 μm filters (Table S1 in [12]). The reads corresponding to this assembly, the reads obtained from sample 6, prefiltered through 3 μm and collected on 0.8 μm filters, and the reads corresponding to the seven other open ocean locations were downloaded from the CAMERA database [45,46]. The *O. tauri *gene content was retrieved from GenBank (accession numbers CR954201-CR954220).

To assess picoeukaryotic diversity, we used eight eukaryotic nuclear gene 'anchors', that is, well-conserved genes across the eukaryotic tree of life: 18S rRNA, 28S rRNA, and genes encoding EF1a, EF2, RPB1, actin, α-tubulin and β-tubulin. For each of the six nuclear protein coding genes, we retrieved the seven corresponding genes from the KOG database [47], corresponding to the genes of *Arabidopsis thaliana*, *Caenorhabditis elegans*, *Drosophila melanogaster*, *Homo sapiens*, *Saccharomyces cerevisiae*, *Schizosaccharomyces pombe*, *Encephalitozoon cuniculi *and the corresponding *O. tauri *gene. We then extended each reference dataset by searching GenBank for representatives of these genes in each of the supergroups of the eukaryotic tree of life [26]. The total number of genes in each dataset was 17 (EF1a), 15 (EF2), 21 (RPB1), 22 (actin), 20 (α-tubulin) and 20 (β-tubulin).

We also used one chloroplast gene, that encoding the large subunit of ribulose carboxylase (*rbcL*), and one mitochondrial gene, that encoding the first subunit of cytochrome oxydase (*cox1*). For each gene, we retrieved 21 and 31 genes from GenBank, respectively, randomly sampling representatives in each of the five supergroups of the eukaryotic tree of life.

To assess prokaryotic diversity on the same dataset, we used 16S rRNA. The reference dataset for 16S rRNA was retrieved from the RDPII database [48] and contained 4,409 sequences. We randomly chose one sequence for each sequence sharing the same taxonomic affiliation (given by the first name of the organism, for example, *Persephonella*), which reduced the number of sequences to 906.

The reference datasets for 18S and 28S RNA were retrieved from GenBank using the ACNUC retrieval system [49] excluding sequences from metazoans. As for the 16S rRNA dataset we randomly chose one sequence when several organisms shared the same taxonomic affiliation. We thus obtained a reference dataset of 252 18S and 246 28S sequences.

### Picoeukaryotic diversity and abundance

To assess the diversity and abundance of picoeukaryotes in this dataset, we performed a BLAST search [50] of the ten eukaryotic 'anchor' genes against the SSD, blastn for RNA and tblastn for proteins. We retrieved all Sargasso Sea scaffolds matching these genes with E-values smaller than 10^-14 ^for blastn and 10^-7 ^for tblastn. We then retrieved these SSD scaffolds and performed a BLAST search against GenBank for taxonomic affiliation. We used blastn against GenBank for scaffolds containing one of the two rRNA genes, and blastx against GenBank's protein database for the scaffolds containing one of the eight 'anchor' protein genes. We deduced the taxonomic affiliation of the environmental sequence from the taxonomic affiliation of the BBH when the E-value of the BBH was smaller than 10^-18 ^(blastn) and 10^-10 ^(blastx). Otherwise, we considered it as unknown.

### Phylogeny of rRNA SSD scaffolds

The SSD scaffolds matching a gene of the anchor 18S and 28S datasets, the corresponding anchor gene and the GenBank BBH, were aligned by MAFFT version 5 [51,52] and the alignment was checked by eye with Se-Al v2.0a11 [53]. Ambiguous regions were deleted from the alignment, for a final length of 3,045 bp for the 28S rRNA dataset (90 sequences in total) and 1,374 bp for the 18S rRNA dataset (61 sequences in total).

Most SSD scaffolds are of different sizes, together covering almost all 18S and/or 28S rRNA. These sequence length differences made it difficult to recontruct a phylogenetic tree directly from the whole matrix of aligned sequences. Thus, overlapping subsets of sequences were defined for the maximum possible number of species, given that the aligned sequences were long enough to reconstruct well-supported phylogenetic trees. The trees issued from these datasets will hereafter be named 'subtrees'. They were reconstructed by Bayesian analysis with MrBayes 3.1.2 [54]. The reconstruction used four chains of 10^6 ^generations with the best evolutionary models chosen via hierarchical likelihood ratio test by MrModelTest 2.2 [55,56] (the MrModeltest 2.2 program is distributed by the author, Evolutionary Biology Centre, Uppsala University). The Burnin value was set to 20% of the sampled trees (1% of the number of generations) and only clades with at least 90% posterior probability support were kept as conservative estimates in the final consensus tree. Thirty-one subtrees (28S rRNA) and 23 subtrees (18S rRNA) were constructed.

All subtrees were combined in a supertree with the use of RadCon [57], using matrix representation with parsimony with the Baum [58] and Ragan [59] coding scheme [60,61]. The combined matrix was subjected to a parsimony analysis with the heuristic algorithm implemented in PAUP* [62], using 500 random addition replicates and the tree bisection-reconnection branch-swapping algorithm, holding a maximum of 1,000 trees for each replicate. The 498,000 (28S) and 423,000 (18S) most parsimonious trees obtained were combined in a majority-rule consensus. Supertrees computed from subtrees obtained via Bayesian analysis and maximum likelihood were not significantly different (*p *< 0.01, symmetric-difference test [63], computed with PAUP* 4.0), and only supertrees computed from Bayesian inferred subtrees are presented. To assign a SSD scaffold to a taxonomic group, the branch support of this sequence within a taxonomic group had to be over 80%; otherwise, we assumed that the taxonomic affiliation of the SSD scaffold was unresolved by the supertree topology.

### Phylogenetic position of the SSD *Ostreococcus *like 18S sequence

The 18S rRNA sequences from several *Ostreococcus *strains [30] and the corresponding first blast hit of the *O*.*tauri *18S on the SSD were aligned manually. This alignment was used to build a phylogenetic tree by Bayesian analysis with MrBayes 3.1.1 [54]. The reconstruction used four chains of 10^6 ^generations with the best evolutionary models chosen via hierarchical likelihood ratio test by MrModelTest 2.2 [56]. The best model was Hasegawa-Kishino-Yano (HKY+Γ) for 18S rRNA. Several analyses were independently run from random trees and to assess convergence. The tree was rooted using related prasinophyte taxa: *Bathycoccus*.

### Sequence analysis

For each SSD scaffold, we computed the length; the number of gaps, the distance between gaps and the base composition using home made computer programs (C language). Statistical analysis was performed with R software [64].

To compare the AT frequency between the SSD scaffolds and the AT frequency of the corresponding BBH, we derived the variance, *V*, of the average of the difference in AT frequency between the two sequences, *M*. Under the null hypothesis of no difference in AT composition, *M *follows a normal distribution of mean 0 and variance *V*:

$$V=\frac{1}{n^{2}}\sum_{i=1}^{n}\frac{f_{i}(1-f_{i})+f{'}_{i}(1-f{'}_{i})}{k_{i}}$$

with *n *the number of SSD scaffolds used, *k*_*i *_the length of the alignment over which the AT frequencies of the SSD scaffold, *f*_*i*_, and the corresponding BBH, *f'*_*i*_, was computed.

## Abbreviations

BBH, best blast hit; EF, elongation factor; GOS, Global Ocean Survey; RPB1, large subunit of RNA polymerase II; SSD, Sargasso Sea Database.

## Authors' contributions

GP designed the study and performed data analysis. YD performed phylogenetic analysis. ED provided *Ostreococcus *sequences and helped with data analysis. GP and HM wrote the paper. All authors have read and approved the final manuscript.

## Additional data files

The following additional data are available with the online version of this paper. Additional data file 1 shows the supertree of 28S rRNA, a consensus of 498,000 trees. Additional data file 2 is the supertree of 18S rRNA, a consensus of 423,000 trees. Additional data file 3 is a table listing the models chosen for each subtree with ModelTest.

## Supplementary Material

Additional data file 1 Supertree of 28S rRNA, a consensus of 498,000 trees. Click here for file

Additional data file 2 Supertree of 18S rRNA, a consensus of 423,000 trees. Click here for file

Additional data file 3 Models chosen for each subtree with ModelTest. Click here for file
