# Supplementary figures and images for: Picoeukaryotic sequences in the Sargasso Sea metagenome
Source: Genome Biol. 2008 Jan 7;9(1):R5. doi: 10.1186/gb-2008-9-1-r5 (PMC2395239; doi:10.1186/gb-2008-9-1-r5)

BBH : GenBank Best Blast Hit sequence.

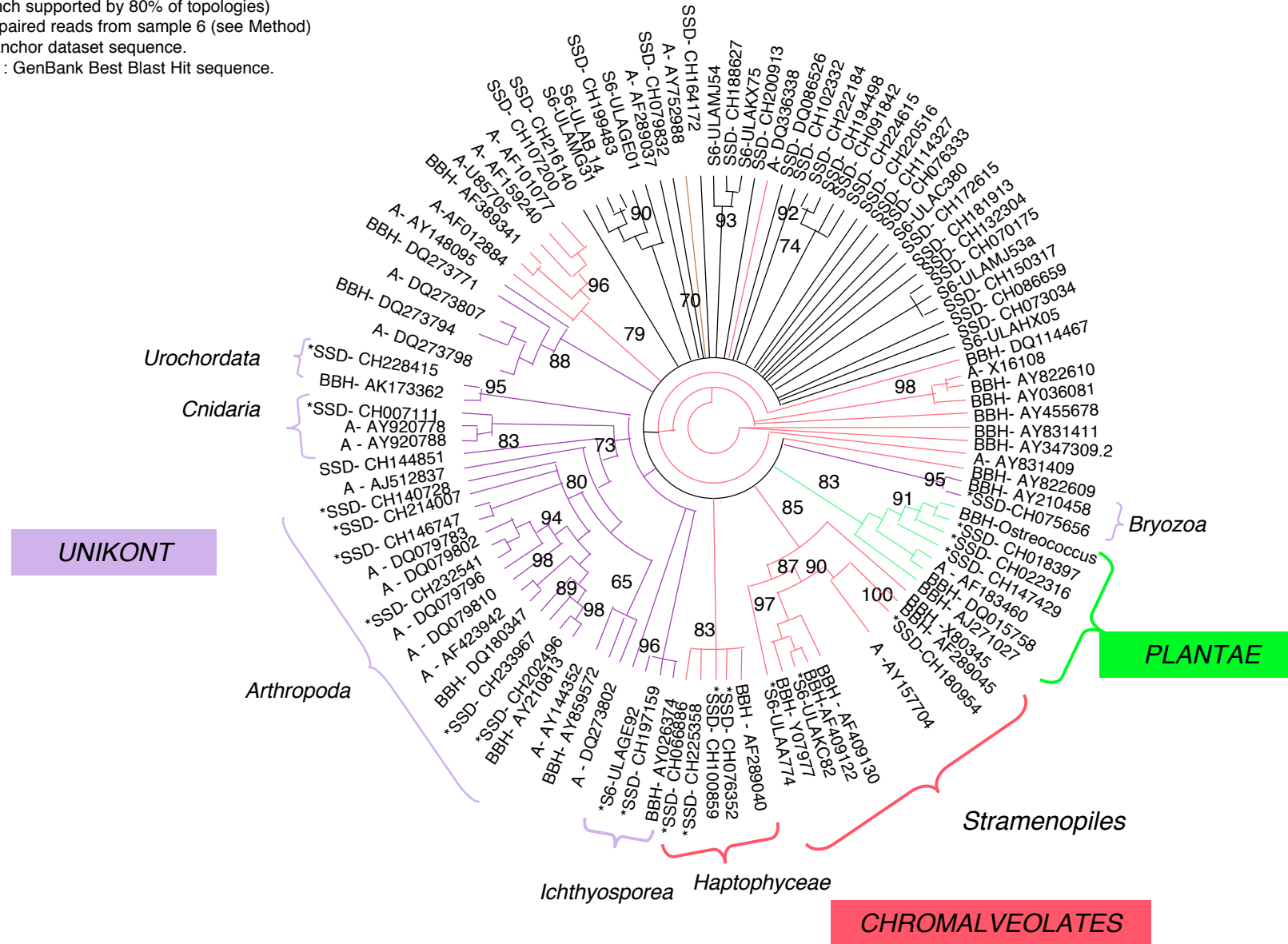

Supplement: Additional data file 1 — Supertree of 28S rRNA, a consensus of 498,000 trees. [file gb-2008-9-1-r5-S1.pdf]
