# Supplementary material for: Picoeukaryotic sequences in the Sargasso Sea metagenome
Source: Genome Biol. 2008 Jan 7;9(1):R5. doi: 10.1186/gb-2008-9-1-r5 (PMC2395239; doi:10.1186/gb-2008-9-1-r5)

SuppFig 2: Supertree of 18S rRNA, Consensus of 423000 trees.
Numbers on branches give percent of topologies supporting a branch.
SSD : Sargasso Sea Database scaffold.
S6 : paired read from sample 6 (see Method).
*SSD : SSD scaffold for which topology enables to assign taxonomic affiliation
(branch supported by 80% of topologies)

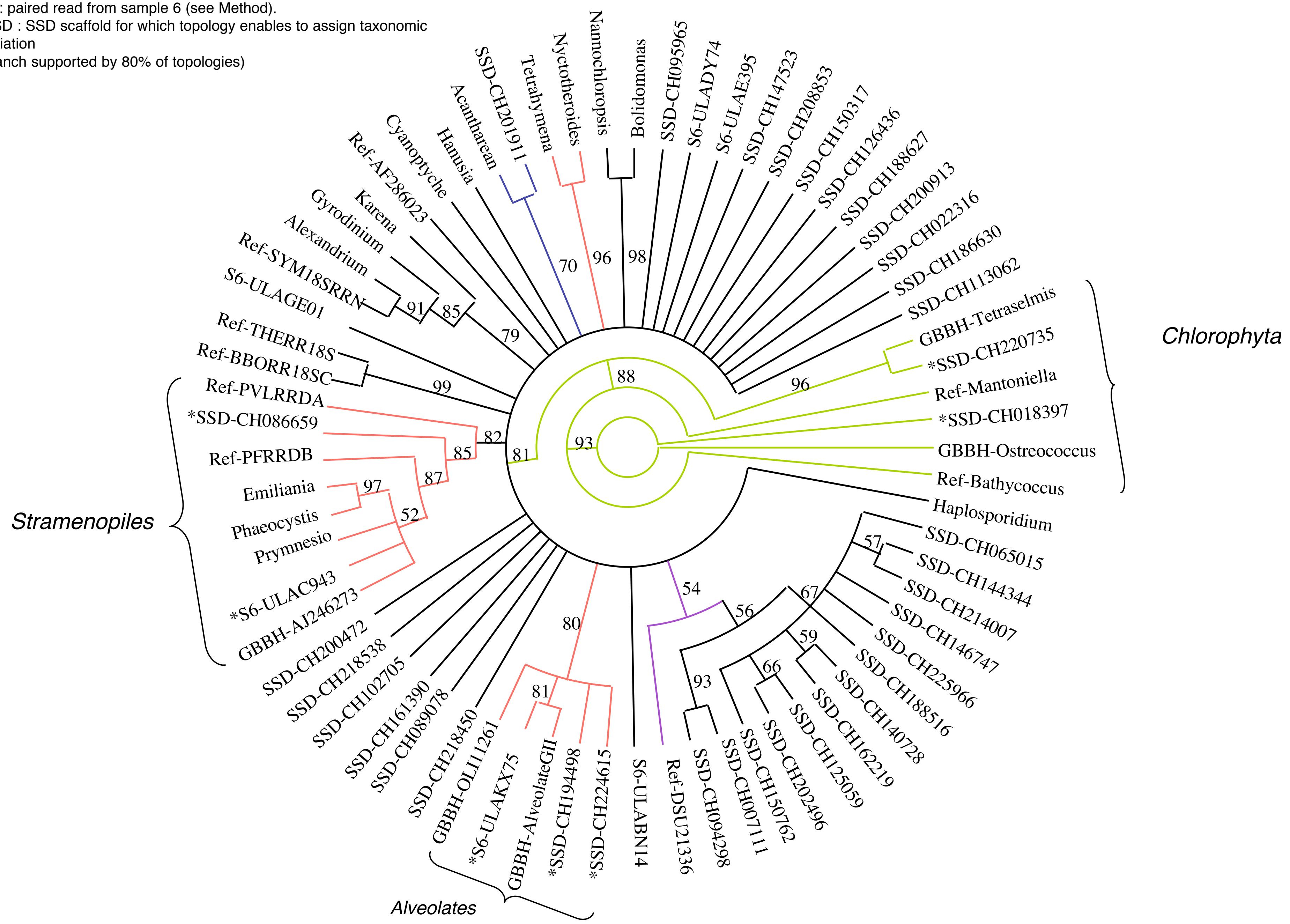

Supplement: Additional data file 2 — Supertree of 18S rRNA, a consensus of 423,000 trees. [file gb-2008-9-1-r5-S2.pdf]
