# Supplementary material for: Picoeukaryotic sequences in the Sargasso Sea metagenome
Source: Genome Biol. 2008 Jan 7;9(1):R5. doi: 10.1186/gb-2008-9-1-r5 (PMC2395239; doi:10.1186/gb-2008-9-1-r5)
Supplement: Additional data file 3 — Models chosen for each subtree with ModelTest. [file gb-2008-9-1-r5-S3.doc]

**Supplementary data file 3**

Models selected with MrModelTest for each subtree reconstructed via Bayesian inference (a) 18S rDNA dataset; b) 28S rDNA dataset). Legend for DNA substitution models: GTR: General Time Reversible, HKY: Hasegawa-Kishino-Yano 1985, K80: Kimura 1980, SYM: Symmetrical, with Γ: shape parameter of the gamma distribution accounting for substitution rate heterogeneity, and I: proportion of invariable sites

a)

| Tree | Number of taxa | Alignment length (bp) | Selected model |
| --- | --- | --- | --- |
| 1 | 6 | 610 | HKY + Γ |
| 2 | 26 | 112 | GTR + Γ |
| 3 | 13 | 626 | GTR + Γ |
| 4 | 19 | 468 | K80 + Γ |
| 5 | 21 | 407 | HKY + Γ |
| 6 | 24 | 171 | HKY + Γ |
| 7 | 12 | 1293 | GTR + I + Γ |
| 8 | 27 | 100 | K80 + Γ |
| 9 | 28 | 1224 | GTR + I + Γ |
| 10 | 39 | 312 | K80 + Γ |
| 11 | 31 | 633 | GTR + Γ |
| 12 | 28 | 798 | GTR + I + Γ |
| 13 | 33 | 641 | GTR + I + Γ |
| 14 | 38 | 314 | GTR + I + Γ |
| 15 | 31 | 659 | GTR + I + Γ |
| 16 | 31 | 665 | GTR + Γ |
| 17 | 37 | 330 | HKY + Γ |
| 18 | 40 | 148 | GTR + Γ |
| 19 | 33 | 419 | GTR + Γ |
| 20 | 30 | 343 | K80 + Γ |
| 21 | 32 | 287 | K80 + Γ |
| 22 | 34 | 236 | K80 + Γ |
| 23 | 41 | 136 | HKY + Γ |

b)

| Tree | Number of taxa | Alignment length (bp) | Selected model |
| --- | --- | --- | --- |
| 1 | 30 | 2396 | GTR + I + Γ |
| 2 | 31 | 1392 | GTR + I + Γ |
| 3 | 36 | 377 | GTR +2Γ |
| 4 | 34 | 378 | GTR + Γ |
| 5 | 37 | 274 | SYM + I + Γ |
| 6 | 60 | 767 | SYM + I + Γ |
| 7 | 61 | 657 | SYM + I + Γ |
| 8 | 66 | 882 | SYM + I + Γ |
| 9 | 70 | 725 | SYM + I + Γ |
| 10 | 74 | 174 | HKY + Γ |
| 11 | 28 | 1598 | SYM + I + Γ |
| 12 | 73 | 285 | GTR + I + Γ |
| 13 | 48 | 1389 | SYM + I + Γ |
| 14 | 51 | 682 | SYM + I + Γ |
| 15 | 28 | 811 | SYM + I + Γ |
| 16 | 52 | 483 | SYM + I + Γ |
| 17 | 47 | 1182 | SYM + I + Γ |
| 18 | 51 | 666 | SYM + I + Γ |
| 19 | 58 | 182 | K80 + I + Γ |
| 20 | 49 | 800 | SYM + I + Γ |
| 21 | 55 | 614 | SYM + I + Γ |
| 22 | 48 | 742 | SYM + I + Γ |
| 23 | 45 | 871 | K80 + I + Γ |
| 24 | 58 | 514 | K80 + I + Γ |
| 25 | 46 | 854 | SYM + I + Γ |
| 26 | 34 | 930 | SYM + I + Γ |
| 27 | 48 | 657 | SYM + I + Γ |
| 28 | 30 | 699 | SYM + I + Γ |
| 29 | 36 | 523 | K80 + I + Γ |
| 30 | 39 | 376 | SYM + I + Γ |
| 31 | 42 | 108 | K80 + Γ |
